# Parricide, Mental Illness, and Parental Proximity: The Gendered Contexts of Parricide in England and Wales

**DOI:** 10.1177/10778012221077127

**Published:** 2022-04-12

**Authors:** Caroline Miles, Rachel Condry, Elspeth Windsor

**Affiliations:** 1 5292 University of Manchester, Manchester, UK; 2 Centre for Criminology, 6396 University of Oxford, Oxford, UK

**Keywords:** parricide, matricide, femicide, parental proximity, mental illness

## Abstract

Parricide is underresearched in the UK, and the contexts of this gendered form of violence are poorly understood. Heide’s typology provides an advanced understanding of parricide in the United States, where the majority of parent-killings involve firearms. This article develops a UK-based analysis of the contexts of parricide, combining national statistics with police case study data (*n* = 57) and case review data (*n* = 21). Our findings indicate that mental illness plays a key role, combined with a gendered context of “parental proximity” and the simultaneous responsibilization and marginalization of parent-victims (particularly mothers), supporting the need for feminist analyses of parricide.

## Introduction

Parricide refers to the killing of a close family member but is commonly used to describe fatal violence from children (of all ages) toward their parents. This form of domestic homicide is relatively rare across the globe, but due to its nature, tends to attract media attention, with cases involving young offenders, female perpetrators, and multiple victims most likely to be sensationalized or exaggerated (Boots & Heide, 2006; Heide, 2013a). In the UK, the most well-publicized case of parricide, which attracted a great deal of media attention and has been widely written about and televised, is the case of Jeremy Bamber, who in 1985, aged 25, killed his adoptive parents, sibling, and nephews, and was convicted for voluntary manslaughter. In the United States, the case of the Menendez brothers, convicted in the 1990s of killing their parents with a shotgun, received a very high level of media attention and is still vivid in public consciousness.

Perhaps due to its infrequency, parricide has not been extensively researched or written about from an academic perspective. The most prominent body of criminological literature derives from Heide’s seminal work in the United States over the past 30 years (see e.g., Heide & Petee, 2007, 2007a; Heide, 1992, 2013a, 2013b, 2014, 2017), through which she has developed a typology of parricide perpetrators: the severely abused adolescent, the severely mentally ill parricide offender, the dangerously antisocial parricide offender, and the enraged parricide offender (Heide, 2013a; Heide, 2017). Outside of this work, much of the literature, until very recently, has come from the disciplines of psychology and psychiatry and has been based on clinical samples, typically convicted homicide offenders serving sentences in secure hospitals (e.g., Baxter et al., 2001; Dantas et al., 2014; Liettu et al., 2014; Marleau et al., 2003). There are a number of limitations with existing knowledge, including the lack of culturally relevant UK-based research, as much of the literature derives from the United States, where the vast majority of parricides involve firearms, many of which are perpetrated by adolescents; an emphasis on clinical samples; and a host of methodological issues, such as variations in definition, study parameters, and small sample sizes.

Notwithstanding this, in recent years, there has been a handful of criminological publications focusing on (or covering, as part of a broader domestic homicide focus) parricide in the UK, including Holt’s (2017) analysis of Homicide Index (HI) data on parricide, Bows’ (2019) analysis of domestic homicide of older people in the UK, and Chantler and colleagues’ (2020) analysis of domestic homicide reviews (DHRs). There have also been two reports on the Femicide Census in the UK, which have included sections on son–mother killings (Brennan, 2016; Long & Harvey, 2020), and in September 2020 a report was launched by the national support organization Standing Together, presenting a case analysis and review of 84 DHRs, which included 17 parricide cases (Montique, 2019). Most recently, Bojanic et al. (2020) published findings from a psychiatry-focused study drawing upon HI data (1997–2014), police national computer data, and mental health service data, providing a comprehensive quantitative analysis of parricide in England and Wales. What this growing body of literature illustrates is that while parricide is relatively low in annual numbers, it remains a persistent and potentially preventable form of homicide. Moreover, this highly gendered form of violence feeds into and overlaps with a number of prominent social problems, including family violence, violence against women, elder abuse, and femicide.

However, we still know little about the qualitative contexts and pathways leading to fatal violence toward parents in the UK and the lived experiences of parricide victims remain untold. The government HI collates data on homicide in England and Wales and provides some indication of circumstances surrounding parricide events, but as we consider below, the depth and validity of data are limited. According to Holt’s (2017) analysis, approximately one-quarter of parricide events involve “irrational acts” (interpreted as denoting an “insane” or “disturbed” perpetrator), leading to the assertion that, contrary to research based on clinical samples, most parricides are *not* a result of mental illness. However, research examining domestic homicide and femicide in the UK through analyzing statutory DHRs has reported high levels of mental illness in domestic homicide perpetrators, including among the small number of parricide cases included in these reviews (Benbow et al., 2019; Chantler et al., 2020; Montique, 2019; Sharp-Jeffs & Kelly, 2016). This concurs with international psychiatric literature on parricide, which reports mental illness, and schizophrenia in particular, to be a key factor in explaining fatal violence from adult-aged children toward parents (Baxter et al., 2001; Bourget et al., 2007; Cantanesi et al., 2015; Liettu et al., 2009; Marleau et al., 2006). Bojanic et al.’s (2020) psychiatric-focused study on parricide in England and Wales also found high levels of mental illness, with 67% of perpetrators having a previous diagnosis, one-third of which involved schizophrenia or another delusional disorder.

The key aim of this article is to develop existing knowledge surrounding parricide in the UK and to begin to construct an understanding of the contexts in which fatal violence toward parents takes place. This builds upon our previous work examining nonfatal violence from adolescents toward parents (see Condry & Miles, 2012, 2015, 2016, 2021; Miles & Condry, 2014, 2015), which is also highly gendered. Within this overarching aim, we explore the prominence of mental illness as a factor in parricide events, given the contradictions in recent UK-based research—and crucially, examine the contextual nature of the apparent relationship between mental illness and parricide. As we will argue, a feminist sociological analysis opens up the deeper contextual familial circumstances of individual cases and also enables exploration of the wider processes that shape parricide and how it is experienced. As DeKesedery has argued in this journal (2021, p. 622), feminist analyses that prioritize patriarchy and gender prompt us to ask questions about how violence against women is closely connected to patriarchy, how society’s gender norms contribute to high rates of violence against women, and how the differential power of men and women might contribute to the problem. Although there are roughly equal numbers of mothers and fathers killed by a son/daughter, parricide is far from a gender-neutral phenomenon and is overwhelmingly perpetrated by men, with significant differences between matricide and patricide victims in terms of the circumstances of the parricide and the histories and life trajectories, informed by gender norms, differential power, and patriarchal structures that particularly marginalize mothers.

The article begins with a review of existing literature on parricide, highlighting the limitations and articulating the need for a more detailed, culturally relevant, and context-specific understanding that prioritizes the analysis of gender. We then briefly present findings from our recent analysis of HI data, before drawing upon the analysis of 57 parricide cases (from one large police force in England and Wales), which enables us to explore the deeper contexts of events. Finally, we draw upon a subset of 21 cases (from our police dataset) for which we were able to access full case reviews, providing detailed histories and facilitating a more contextualized insight into the pathways and mechanisms connecting mental illness with the killing of parents.

## Understanding Parricide

Although large-scale national empirical analyses of parricide are rare, research across the globe consistently finds that parricide is an infrequent event (Bojanic et al., 2020; Bourget et al., 2007; Heide, 2013a; 2013b; Heide & Petee, 2007a; Holt, 2017; Shon & Targonski, 2007; Walsh et al., 2008). In England and Wales, the number of parricide events fluctuates year on year, but averages just under 20 per year (Bojanic et al., 2020; Holt, 2017). As a proportion of all fatal violence, parricide accounts for 2% of all homicide in the United States (Heide, 2013a), and approximately 3%–4% of all homicide in England and Wales (Bojanic et al., 2020; Holt, 2017). Parricide constitutes 12% of all *domestic* homicide cases in Australia (Benier, 2016) and 9% of all *domestic* homicide in Canada (Bourget et al., 2007).

### Parricide as a Gendered Phenomenon

As with all homicide, the vast majority of parricide perpetrators are male, reportedly accounting for between 85% and 90% of convicted offenders (Bojanic et al., 2020; Heide, 2013a; Holt, 2017; Walsh et al., 2008); and in England and Wales, mostly white (84%), unmarried (89%), and living with parents (70%) (Bojanic et al., 2020). The average age for parricide perpetration appears to be around 31 years (Bourget et al., 2007; Holt, 2017). Parricide is also conceptualized as a gendered form of violence; unlike homicide per se, in which females only constitute 27%–30% of victims (ONS, 2021), the sex of victims in parricide tends to be fairly evenly distributed, meaning that women are at a greater risk of being killed by their offspring (mostly sons) than by someone outside of the family. Bourget et al. (2007) reported that 57.8% of parricides in Canada involved male victims (“patricide”) compared to 42.4% female victims (“matricides”); Heide and Petee (2007) found that 55% of parricide victims in the United States were male, compared to 45% female; and both Bojanic et al.’s (2020) and Holt’s (2017) analyses of parricides in England and Wales revealed a male/female victim ratio of 51:49.

In light of evidence that parricide is a gendered phenomenon, an important question emerges around whether mother killings, or matricides, ought to be considered a form of femicide (the killing of women by men *because* they are women). Relatedly, it seems pertinent to disaggregate patricides and matricides in order to establish whether they represent distinct phenomena; that is, to consider whether mothers are killed in different contexts and circumstances to fathers. To this end, Holt (2017) found that the risk of parricide victimization changes across the lifecourse and is different for mothers and fathers—*patricide* victimization in England and Wales is most likely to occur when fathers are in their 50s, whereas the risk of *matricide* victimization increases with age and peaks for mothers in their 70s. Bows’ (2019) analysis of domestic and family homicide among older people also found that older women (aged over 60) are more at risk of being killed by their child compared to older men. Similarly, in the United States, Heide (2013a) reported that patricide victims averaged 56 years of age, whereas matricide victims averaged 60 years; also reporting that between 1976 and 2007, 22% of mothers were killed by their biological children aged over 40 years, compared to only 11.9% of fathers. These findings support Heide and Petee’s (2007) earlier report that the mean age of patricide perpetration was lower than for matricide perpetration (respectively, 25 and 30 years). Combined, these cross-national data indicate that fatal violence toward fathers may occur in contextually distinctive circumstances compared with fatal violence toward mothers.

### Cross-Cultural Differences in Parricide

Research examining the incident characteristics of parricide events emphasizes the need for country-specific research, in order to produce culturally relevant knowledge and understanding. In the United States, for example, firearms account for a vast proportion of all homicides; 54.9% of parricides analyzed by Heide and Petee (2007a) involved a firearm (accounting for 64% of patricides and 44% of matricides); and they feature particularly highly in cases of parricides perpetrated by juveniles (under age 18 years): 80% of patricides and 62% matricides. In England and Wales, however, firearms are rarely used in homicides, and in parricides in particular (accounting for approximately 2% of all parricides), with the predominant method of killing recorded as a sharp instrument (49%) (Bojanic et al., 2020).

This important disparity raises questions over the applicability of dominant explanations of parricide emanating from the United States to other countries and highlights the importance of locally sensitive contextual data. Context also extends beyond incident characteristics to the histories and life trajectories before a parricide and may also indicate important cultural, national, and local differences such as family structures and dynamics, patterns of caring, welfare and health provision, criminal justice responses, and definitions of domestic abuse and responses to it, all of which require “local” explanations that facilitate the identification of continuities and discontinuities across jurisdictions.

### Pathways to Parricide

Heide (2013a, 2013b, 2014; Heide & Petee, 2007, 2007a) has produced a significant body of literature on parricide, particularly focusing on adolescent perpetrators of parricide in the United States. Her theoretical insights draw upon existing research, and over 30 years of working with perpetrators of parricide in her capacity as a licensed mental health professional. Heide’s typology (2013a; 2017) asserts that there are four types of parricide offender. The first is the “severely abused adolescent,” which Heide argues is the most common explanation for adolescent parricide offenders. Second, the “severely mentally ill parricide offender” is said to account for the majority of adult parricide perpetrators. The third type of parricide offender identified is the “dangerously antisocial parricide offender,” who often has a history of antisocial behavior, a diagnosed personality disorder, and kills for “selfish reasons.” And the fourth type, proposed initially in 2013 but further developed in 2017 (Heide, 2017), is the “enraged parricide offender,” who kills their parent in the context of long-term child–parent conflict, often in circumstances involving alcohol and/or drug intoxication, and potentially following historic child abuse (Heide, 2017).

Bojanic et al.’s (2020) typology of parricide in England and Wales found some support for Heide’s US-based typology, reporting that 40% of perpetrators were classified as “severely mentally ill,” 42% were “previously abused as children,” and 18% were “middle-aged with affective disorder,” referring to a group of mentally ill perpetrators predominantly aged 45–65 years, who had taken on the role of carers for their aging parents. Although this final category was distinguished by the authors from Heide’s third category of “dangerously antisocial personality disorder,” it is worth noting that Heide (2013a; 2017) also observed parricides occurring in the context of middle-aged and mentally ill sons/daughters who were caring for their aging parents. These findings indicate that despite cultural differences between the UK and US, there appear to be clear similarities in the circumstances leading to parricide.

### The Role of Mental Illness in Parricide

The typologies constructed by Heide (2013a) in the United States, and Bojanic et al. (2020) in England and Wales provide valuable insights into the potential contexts of fatal violence toward parents and indicate that mental illness plays a significant and causative risk factor. Their work is supported by international psychiatric research, which although limited due to relying on small, clinical samples that potentially overreport the role of mental illness, also find that severe mental illnesses including schizophrenia and other delusional disorders are common among parricide perpetrators (Baxter et al., 2001; Bourget et al., 2007; Cantanesi et al., 2015; Green, 1981; Liettu et al., 2009; Marleau et al., 2006). What is currently lacking however is any understanding of the mechanisms by which these mental illnesses may lead to the killing of parents, and the gendered contextual circumstances surrounding these parricides.

Until recently, there has been no criminological research in England and Wales providing an understanding of the pathways leading to fatal violence toward parents; knowledge on parricide has been limited to the disciplines of psychology and psychiatry. In 2017, Holt published the first criminological article specifically examining parricide, presenting a descriptive analysis of HI data on child to parent killings spanning 1977–2012 (including children aged <18 and adult-aged children). Although limited in depth due to the data available, Holt’s analysis examined the situational circumstances of parricide events, reporting that 14% of perpetrators were recorded as intoxicated by alcohol at the time of the killing (although the accuracy of the HI data on intoxication has been criticized, see Miles, 2012); 60% of fatalities were caused by a sharp or blunt instrument; and in 46% cases, the circumstances surrounding events were recorded by police as “other” or “circumstances not elsewhere specified,” highlighting the inadequacy of existing HI categories for mapping out the contexts of parricide.

On the role of mental illness in parricide cases, Holt (2017) found that almost one quarter (24%) of incidents were recorded as an “irrational act,” which was interpreted as denoting mental disturbance, and noted that this category was used more frequently in cases involving female victims (35%) compared to male victims (14%)—again indicating that matricides and patricides are distinctive phenomena and that mental illness is more likely to contextualize fatal violence toward mothers. Twenty-four percent of cases successfully involved the defense of “diminished responsibility,” and 31% of convicted offenders were sentenced to a hospital order. These findings led Holt to question the assumed predominance of mental illness as a context of parricide, highlighting that “most parricides are *not* the product of mental illness: the majority of cases were *not* identified as an ‘irrational act,’ were *not* mitigated on the grounds of diminished responsibility and did *not* conclude with the issuing of a Hospital Order” [original emphasis] (2017, p. 14). This followed Holt and Shon’s (2016) earlier caution against overemphasizing the role of mental illness in parricide, highlighting the potential to neglect other preceding contextual factors.

However, we argue that a cautionary note should be made about the potential limitations of relying on a recording of “irrational act,” the successful use of diminished responsibility as a defense, or a hospital order at sentencing, as being indicative of mental illness in parricide offenders, as detailed below. Our caution is echoed by Bojanic et al. (2020), who found significantly higher levels of mental illness among parricide perpetrators (67%) and classified 40% as “severely mentally ill,” with a further 42% classified as “middle-aged with affective disorder.” Bojanic et al. (2020) contend that the proxy variable “irrational act” relied upon in Holt’s (2017) analysis is inadequate, given that it does not reflect a mental illness diagnosis.

We agree with Bojanic’s (2020) concerns about the reliance upon HI data to determine the role of mental illness in parricide, for a number of additional reasons. First, it is likely that the HI underrecords the frequency of “irrational act,” especially given the large proportion of cases in which no specific circumstance is recorded. The HI is a robust source of data and informs official statistics on homicide published by the Office for National Statistics; however, it has long since been recognized that there are inaccuracies and inconsistencies in recording practices, especially in the more historic data (Moxon, 2001). With regard to the use of diminished responsibility, the legal test for this statutory partial defense to murder^1^ requires a number of factors *in addition to* a defendant suffering from mental illness to be present in order for it to succeed. Specifically, it must also be established that the mental illness impaired a defendant’s ability to: a) understand the nature of their conduct, and/or; b) form a rational judgment, and/or; c) exercise self-control.^2^ *And,* that said, impairment(s) “provide an explanation” for the killing.^3^ Relying on the successful use of diminished responsibility as a defense alone therefore potentially risks under-extending understandings of the role of mental illness in parricide by confining considerations to this highly legalistic construct, which can foreseeably sit uneasily with normative perceptions of offender culpability as those suffering from mental illness may fail to satisfy the other elements of the legal test.

Finally, reliance on the imposition of hospital orders at sentencing to measure mental illness risks similar under-extension. Hospital orders are seen somewhat uniquely “as an alternative to punishment”^4^ at sentencing and the circumstances in which they can be used are heavily circumscribed by the requirements of s.37 Mental Health Act 1983. Where a penal element for criminal justice disposal of an offender is judged to be appropriate, guidance is clear that hospital orders should not be used. Consequently, parricide offenders suffering from mental illness may be dealt with by way of custody with a hospital direction,^5^ or simply by custody alone, and not necessarily by way of a hospital order. It is important to recognize that the sentencing considerations relevant to offender mental illness in cases of parricide will inevitably be complex, and subject to acute variation depending on the specific facts of each case.^6^ Accordingly, it is too simplistic to suggest that the absence of a hospital order necessarily correlates with the absence of offender mental illness in cases of parricide. Combined with Bojanic’s (2020) caution about the lack of diagnostic input into the “irrational act” circumstance within the HI data, we argue that there is a need for a review of administrative data on parricide (and homicide, more broadly) so that the important context of mental illness can be more accurately reflected and documented within official narratives of parricide.

In recent years, UK-based research on domestic homicide and femicide using alternative sources of data has provided evidence that many more parricide (and matricide, in particular) cases than recorded in the HI are perpetrated by people with mental illness, and reaffirmed the need to examine parricide through a gendered lens. Brennan’s (2016) report on the Femicide Census covering England and Wales highlighted that in 63% of cases in which sons had killed mothers (a small subset of the femicide cases), there were contextual circumstances involving mental or physical health issues or substance misuse—and the majority of these cases involved mental health issues, either on their own or combined with physical health/substance misuse issues. The more recent report on femicide in the UK in 2018 (Long & Harvey, 2020) also draws attention to the prevalence of mental illness among perpetrators of matricide, as does a recent report on domestic homicide from the organization Standing Together (Montique, 2019). This report also flags the vulnerability of often single and elderly mothers, who care for their mentally ill son/daughter, and urges further research into this gendered aspect of domestic homicide.

Chantler et al.’s (2020) research on domestic homicide analyzed 141 DHRs across England and Wales, 19% of which were matricides. The findings pertaining to risk factors for the broad category of domestic homicide included victims’ mental health difficulties (observed in 29% of cases) and perpetrator mental health problems (observed in 64% of cases), with 49% of all perpetrators having a diagnosed mental illness, most commonly comorbid depression and anxiety. Finally, albeit based on similarly small sample sizes, research by Sharp-Jeffs and Kelly (2016) and Benbow et al. (2019), exploring the contexts of domestic/adult family homicide, has highlighted the prevalence of mental illness among parricide offenders, and importantly, the role of “caring” for a mentally ill (adult-aged) son/daughter as a risk factor for parricide victimization.

This emerging body of criminological work is supported by psychiatric research examining the relationship between mental illness and homicide more broadly. Rodway et al.’s (2009) analysis of homicide across England and Wales between 1997 and 2003 reported diagnostic differences between perpetrators who use different methods of homicide, finding that perpetrators with diagnoses of schizophrenia were significantly more likely to use a sharp instrument and to kill a family member. Oram et al. (2013) found that 10% of all homicide offenders in England and Wales (covering 1997–2008) had symptoms of mental illness at the time of their offense, 34% of “adult family homicide” offenders (213/251 of which were parricides) had symptoms of mental illness at the time of the homicide, 27% had symptoms of psychosis, and 23% had been in contact with mental health services in the year leading up to the event. Their analysis also revealed that 45% of adult family homicide offenders had ever (in their lifetime) received a mental health diagnosis, and 28% had ever received a primary diagnosis of schizophrenia. These findings support broader international homicide research, which has established that mental illness (particularly psychotic disorders and personality disorders) is more prevalent among homicide offenders than the general population, and that homicides by people with psychotic disorders are statistically significantly more likely to involve the killing of blood relatives (e.g., Hakkanen & Laajasalo, 2006).

The picture, therefore, emerging from existing literature on homicide, domestic homicide, and adult family homicide is that mental illness plays an important role in fatal violence toward parents, implying that many parricides may be preventable. However, this somewhat contrasts with the findings from the large-scale national analysis published by Holt (2017), which is the only piece of criminological research specifically and exclusively focusing on parricide in England and Wales and concluded that mental illness does *not* play an important role in parricide. As highlighted earlier, there is very little knowledge and understanding of the antecedents leading to and contexts surrounding parricide in England and Wales, conflicting reports concerning the role of mental illness, and a poor understanding of the mechanisms connecting mental illness with the killing of parents, especially regarding the role of mothers.

With this in mind, our key aims were to begin to construct in-depth knowledge and understanding of the contexts surrounding parricide in England and Wales, to examine parricide through a feminist lens, and to explore the prominence and nature of mental illness as a factor. In the analysis and discussion below, we first present our own brief analysis of recent HI data covering the period of 2003–2016, before delving further into the contexts of parricide through examining 57 cases of parricide recorded within one English police force. Our findings illustrate not only the key role that mental illness appears to play in a substantial proportion of parricide events—and matricide, in particular--but also identify the concept of “parental proximity” as a key contextual mechanism, which derives from the caring role assumed by many parents of adult-aged mentally ill sons/daughters and particularly relates to mother-killings.

## Homicide Index Data (2003–2016)

Homicide data in England and Wales are recorded in the Home Office owned HI, which collates data from the 43 police forces and documents details of the suspect and victim demographics as well as victim-perpetrator relationship, incident characteristics, and court outcomes. HI data are considered to be some of the most detailed and robust data on homicide in the world and are designated official statistics on homicide in England and Wales (ONS, 2021). However, the database is subject to inaccuracy and missing data, and the data are also subject to a degree of inconsistency across forces. There have been a number of changes to recording practices over the years, most recently following a review in 2000 (Moxon, 2001), which led to a number of changes being implemented.

The data on which the analysis below was conducted were obtained from the Home Office in an anonymous Excel spread sheet and converted into an SPSS database for the purposes of analysis. A data cleaning process removed all erroneous and duplicate data held within the raw database. Following this, all homicides recorded as involving a child-to-parent/stepparent relationship were extracted. There were 271 parricide events recorded between April 1, 2003 and March 31, 2016 (approximately 21 per year and representing 5% of all homicide offenses over this period), with a total of 288 parricide victims. There were 41 suspects for which no data were available; therefore, the statistics relating to the suspect characteristics are based upon 249 suspects.

Our analysis revealed that parricide victims were equally likely to be male or female (compared to the females accounting for under one-third of all homicide victims), although a greater proportion of mothers were known to have been victims of violence prior to their death than fathers (14% compared to 7%). The median age of victims was 61 years, and the mean age was 63. However, female victims were older than male victims: The mean age for matricide victims was 66 years, whereas the mean age for patricide victims was 61 years, and this difference was significant (t = 3.672, df = 247, *p* < 0.001). Suspects were overwhelmingly male (88%), with a median age of 31 years old (mean age 34, range 14–69), and 47% of suspects were single, having never been married; 37% of suspects were living with their parent/stepparent victim at the time of the parricide. Female perpetrators were more likely to have killed their mother (61% cases) rather than their father (39% cases), whereas male perpetrators were equally as likely to have killed either parent.

Our analysis also revealed a significant difference (t = −5.138, df = 247, *p* ≤ 0.001) in the age of suspects for matricides (suspect mean age 38 years) versus patricides (suspect mean age 30 years): the age of suspects is generally much higher in matricides than patricides. For example, 35% of fathers were killed by a son/daughter aged 18–25 years, compared to only 16% female victims. These findings suggest that, in accordance with existing research on parricide, the killings of mothers and fathers, and parricides by sons versus daughters, may represent quite distinct phenomena and should be disaggregated in parricide research in order to fully understand how the contexts and pathways surrounding fatal violence toward parents intersect with gender.

As noted earlier, the HI is limited in what it can tell us about the contexts of parricide events, but some insight is gained via the weapon and circumstances variables. With regard to the former, our analysis supported previous UK-based research (Bojanic et al., 2020; Holt, 2017), with the use of a sharp instrument (49%) by far the most common method, and 40% of incidents specifically involved a knife as a weapon. Analysis of circumstances also supported Holt’s (2017) research and highlighted the inadequacy of HI categories. For 40% of cases, the circumstance was recorded as some sort of “domestic dispute” (e.g., involving jealousy), and 29% of victims were recorded as being killed as a result of an “irrational act,” implying that the suspect was mentally disturbed at the time of the event. Almost one-third (31%) of suspects had been drinking and/or taken drugs at the time of the killing, and 22% had a history of illegal drug use.

Finally, the data revealed that 34% of suspects were convicted of manslaughter on the basis of diminished responsibility (77% of whom received hospital orders). Overall, 39% of parricide offenders convicted for offenses between 2003 and 2016 were given a hospital order, compared to only 4% of all homicide offenders. Fifty-three percent of parricide offenders were given a prison sentence, compared to 89% of all homicide offenders. Although these figures are not substantially different than those cited from Holt’s (2017) analysis above, which covered a longer period of time (1977–2012); our analyses show that in this more contemporary time period (2003–2016), higher proportions of parricides were officially recorded as involving a context of mental illness. This could be due to the development of more accurate recording practices or reflect a growing prevalence of mental illness as a factor in fatal violence toward parents. However, as discussed earlier, we are skeptical as to the extent to which these data provide a valid or reliable measurement of mental illness.

## Contexts of Parricide: Case Study Analysis

In light of the limitations of the HI data, we supplemented national-level data with a case study analysis of 57 parricides recorded between 2002 and 2017, within one large police force area in England. The original case details (offender/victim demographics, incident details, case outcomes where available) were provided to us by the police force, and these were used as the basis of a more detailed search for case study data, containing as much detail as possible about the circumstances and contexts of each event. This included searches of case law reports (using Westlaw, Lexis, HM Courts and Tribunals); DHRs;^7^ Independent Investigations into Mental Health Homicides^8^ (IIMHHs) reports; Safeguarding Adult Reviews^9^ (SARs); local and national media; and general, web-based searches using engines such as Google and Microsoft Bing. The data and level of detail available varied enormously from case to case but allowed for the further development of the police data and for detailed and layered case studies to be constructed for many of the cases. For 21 of the 57 cases, we were able to access a DHR, IMMHH, SAR, or Court Report, which provided in-depth data about the contextual pathways to parricide (drawn upon below, under “Parental Proximity”).

The 57 cases analyzed included 55 single-parent homicides (i.e., one parent was killed) and two double-parent homicides (where both parents were killed), giving a total of 59 parricide victims. Our analysis focused on the killing of biological parents only, due to our interest in the child–parent relationship, and concern about stepparent cases confounding the data, as cautioned by Heide and Petee (2007), and so cases involving stepparents or parents in-law were removed. Of the 59 victims in our case study analysis, 58% (*n* = 34) of the parent victims were mothers, and 42% (*n* = 25) were fathers, a gender split reflecting a slightly larger than average proportion of matricides compared to national-level analyses. The perpetrator analysis revealed that 53 of the 57 parricide incidents (and 55 out of 59 victims) were committed by sons (93%), and just four by daughters (7%); one of whom was recorded as a transgender woman. Given this very small number of female perpetrators, it was not deemed useful to disaggregate the perpetrator analysis by gender. However, we did disaggregate the data by victim gender in order to further explore differences between matricide and patricide. A summary of our key findings is provided in Table 1. As discussed below, a number of differences were observed between matricides and patricides, although these differences were not significant, which could be due to the small sample size.

**Table 1.** Summary of Key Findings.

	Matricides (*n* = 34)	Patricide (*n* = 25)	All Parricides (*n* = 59)
Perpetrator Son	31 (53%)	24 (41%)	55 (93%)
Perpetrator Daughter	3 (5%)	1 (2%)	4 (7%)
Perpetrator Mean Age	39	34	37
Victim Mean Age	69	66	68
Sharp Instrument	21 (62%)	13 (52%)	34 (58%)
Perpetrator Mentally Ill	26 (77%)	17 (68%)	43 (73%)

Analysis of the victim-perpetrator relationship showed that 53% (*n* = 31) of parricides involved a son–mother relationship; 41% (*n* = 24) involved a son–father relationship; 5% (*n* = 3) involved a daughter-mother relationship; and just one parricide (2%) involved a (transgender) daughter–father relationship. Although this sample of 57 cases is limited in its generalizability due to the small number of cases from a single force, and thus restrictive in terms of meaningful statistical analyses, the data support national analyses that have illustrated the highly gendered nature of parricide.

The age of parricide perpetrators ranged from 15 to 61 years, with a mean age of 37 years. Only one perpetrator in this sample was a juvenile offender (15 years old) and one perpetrator was aged 18 years; all remaining perpetrators were aged over 20 years. The age of the victims ranged from 43 to 100 years of age, with a mean age of 68 years. The mean age of matricide victims was 69 years (range 43–100 years), which was slightly higher than patricide victims, who had a mean age of 66 (range 47–84 years), although this difference was not significant. Notably, only 8% (*n* = 3) of matricide victims were under the age of 50, compared to 20% (*n* = 5) of patricide victims, providing further evidence that the risk of parricide varies by gender across the lifecourse. The recording of ethnicity of victims indicated a disproportionate number of nonwhite victims of parricide. Only 39% of this case study sample were recorded as White (British/European), 23% as Black British/African/Caribbean, and 23% Asian. This compares to a population breakdown for the area of approximately 60% white, 13% Black British/African/Caribbean, and 18% Asian. This disproportionality, which has not been highlighted elsewhere (see e.g., Heide, 2013b; Heide, 2014), could reflect poor data accuracy for ethnicity, or it might indicate important cultural differences requiring further examination.

Police data on method of parricide revealed that the use of a “sharp instrument” was by far the most common, accounting for the death of 58% (*n* = 34) of victims, followed by “blunt instrument” (15%, *n* = 9), “strangulation” (12%, *n *= 7), and “suffocation” (7%, *n* = 4). In contrast to parricide in the United States, where guns are used in a significant proportion of parricides, there were no parricides involving a shooting in this sample. When disaggregated by gender, some differences were apparent in the methods of killings for mothers compared to fathers, providing further evidence of matricide and patricide being distinctive phenomena: 62% of mothers (*n* = 21) were killed using a sharp instrument, compared to only 52% of fathers (*n* = 13); However, the use of other methods of parricide was similar for both mothers and fathers (e.g., 15% of mothers and 16% of fathers were killed using blunt instruments), and the overall differences were not significant.

One of the most striking findings of the perpetrator analysis related to the prevalence of mental illness among this sample of 57 parricide cases. In total, our deeper contextual analysis found evidence of mental illness diagnoses for 74% (*n* = 42) of the 57 parricide offenders [and 43 (73%) of the 59 parricide victims were killed by their mentally ill son or daughter]. For 79% of these offenders (*n* = 33, 58% of the total sample), the mental illness was found to be *legally causative* of the killing (this is elaborated on below), leading to a conviction for manslaughter due to diminished responsibility, rather than murder. In 7 of the 42 cases involving mental illness (17%, or 12% of the total sample), the mental illness was not found to be legally causative, and in 2 cases, the perpetrator committed suicide and so there was no trial.

When broken down by gender, over three-quarters of all matricides (77%, *n* = 26 of 34 female victims) in this sample were committed by a mentally ill son or daughter, compared to two-thirds of all patricides (68%, *n* = 17 of 25 male victims), a strikingly high number (although this difference was not significant). These findings unequivocally resound with recent research examining the prevalence of mental illness as a significant factor in domestic homicide (especially adult family homicides/parricides) and support our earlier caution about relying on homicide statistics from the HI database to account for mental illness. They also lend further support to the need to examine parricide through a gendered lens and considering matricides and patricides as distinctive.

Finally, there was some evidence of previous child abuse in just 7% of the parricides: three son–father killings and one son–mother killing indicated a history of child abuse. This is significantly lower than the 42% of parricide perpetrators reported to have been previously abused in Bojanic et al.’s (2020) analysis, although child abuse was only found to be the motive in 11% of cases. It also contrasts with Heide’s typology, which includes a category of (juvenile or young adult) parricide offenders who were abused as children and kill their abusive parent(s) as their only perceived means of escape. This discrepancy could reflect the small size of our case study sample, which only included one perpetrator aged under 18. However, we also assert that it is reflective of the poor quality of available data on parricide, and the differential agendas of data sources that are not constructed with research in mind. Bojanic et al.’s (2020) data on case histories derived from reports by mental health services with whom perpetrators had been in contact in the 12 months preceding the parricide. These data will understandably be perpetrator-focused and detailed in terms of documenting their personal histories and potential reasons for killing their parents. Police data, on one hand, are more concerned with recording evidence against the suspect, and DHRs, on the other hand, are more victim-focused and less concerned with potentially justifying perpetrators’ behavior. This resonates with Cullen et al.’s (2021) recent recognition of the limitations of administrative data (including official statistics and DHRs), and the need for more rigorous and quality data recording, with research and prevention purposes in mind.

### Perpetrator Mental Illness

As documented above, in the overwhelming majority of cases in this sample of 57 parricides from one English police force, the police case files contained references to mental health problems, diagnoses, and legal defenses, many of which were upheld in court. In a number of cases, the police notes stated that the suspect was deemed unfit to interview due to their mental state, which indicates that there were mental health problems at the time of the event, or that the defendant was experiencing mental health problems as an immediate result of killing their parent. In other cases, there was clear evidence that the perpetrator had a prior mental illness diagnosis.

It is important to recognize that in discussing the mental illness of perpetrators, correlation is not necessarily causation. To understand the degree to which mental health crises of offenders can fairly be understood to be causative in these killings, this research undertook a careful analysis of the data sample to distinguish cases where the presence of offender mental illness was found to be both factually and legally causative in the killing by the courts and those that were not. To this end, we use the term “legally causative” to denote mental illness being relied upon by the court in some way when establishing the causation of the killing, such as for example, through successful use of diminished responsibility as a defense; the successful use of insanity as a defense; instances where the defendant was deemed unfit to stand trial/plead as a result of mental ill-health, which had also been operative at the time of the killing; the coroner attributing killing to the now deceased offender’s mental health crisis; or the defendant pleading to manslaughter due to the *mens rea* (intent) being impacted by the defendant’s mental illness.

These cases were distinguished from cases where the data indicated that offender mental illness may be considered to be “factually causative” in that the defendant was recognized as suffering from mental illness at the time of killing but was unable to align this with a legal defense or with evidence of reduced culpability/lack of intent. It is likely therefore that the findings of this analysis sit on the conservative end of the spectrum when considering the causative potency of perpetrator mental illness in parricide killings, and possibly, as with the predecessors of literature in this field, under-extend the relevance of perpetrator mental illness in parricide.

By far the most prevalently diagnosed mental illness in this sample was schizophrenia, followed by other delusional disorders. This concords with clinical research examining the relationship between schizophrenia and violent behavior (Jovanovic et al., 2019) and a meta-analysis by Fazel et al. (2009), which concluded “there is a robust body of evidence that demonstrates an association between the psychoses and violence” (p. 12). Although schizophrenia was overwhelmingly the most common psychiatric illness in the data sample, it was by no means the only one and there were other cases where different psychiatric illnesses were found to be both factually and legally causative in homicide, including, for example, depressive disorders and comorbidity with alcohol and/or drug misuse.

Broadly speaking, our findings in relation to the prevalence of mental illness among parricide perpetrators within this sample of 57 cases supports Bojanic et al.’s (2020) analysis of parricide in England and Wales, and UK-based research on domestic homicide and adult family homicide (incorporating parricide) (Benbow et al., 2019; Chantler et al., 2020; Oram et al., 2013; Rodway et al., 2009; Sharp-Jeffs & Kelly, 2016), as well as international psychiatric research highlighting the frequent co-occurrence of mental illness (especially schizophrenia) and fatal violence toward parents (Baxter et al., 2001; Bourget et al., 2007; Cantanesi et al., 2015; Green, 1981; Liettu et al., 2009; Marleau et al., 2006). Importantly though, co-occurrence does not necessarily denote a causal relationship, and as highlighted by Hillbrand et al. (1999) and Cantanesi et al. (2015), it is pertinent to examine the broader contexts underpinning this association.

### Parental Proximity

In order to further explore the role of mental illness and understand the mechanisms that appear to link severe mental illness with parricide, we turn to a subset of 21 cases (extracted our sample of 57 parricide cases) for which we were able to obtain full reviews of some kind, for example, from DHRs, Independent Investigations into Mental Health Homicides (IIMHHs) reports, and Safeguarding Adult Reviews (SARs), which provided a detailed case history. These reviews revealed that in 20 of the cases, the perpetrator had a serious mental illness, including schizophrenia and other delusional disorders. What was particularly striking about these cases, however, was the theme of “parental proximity,” referring to the high number of cases (*n* = 19) within this subset where there were dependent caring relationships, in most cases, involving mothers who were killed by their sons.

In 14 cases, the parent-victim had been the primary caregiver for their mentally ill son in the lead up to their death, providing physical, emotional, and financial support. Of these cases, 10 were son-to-mother matricides where the mother had been the primary carer for the perpetrator. Four were son-to-father patricides, but it is notable that although it was the father who was killed, in these cases the son was being supported by both his father and his mother prior to the homicide. In most cases, the perpetrator lived with their parents, but even those who lived independently relied heavily on their parents for day-to-day care and support.

In all ten matricide cases, the mothers were primary carers providing a wide range of practical and emotional support including providing a home, financial support, managing mental health symptoms, organizing health care, and support with a range of aspects of daily living. None lived with partners, and they were often isolated in providing care to their sons. These mothers were marginalized in the treatment of their sons despite being their primary carer. In some cases, they were ignored, even when they raised concerns about their son and the risk he might pose. In some cases, there were recorded examples of previous violence that was not recorded as domestic violence or responded to appropriately. In others, troubling signs were ignored. Overwhelmingly, the mothers’ own needs were disregarded, and their vulnerability and risk were not assessed.

In a further five cases, the perpetrator was caring for their parent-victim prior to the killing, providing practical care for what were primarily physical health care needs because the parent was very elderly, had suffered a stroke, or had dementia. Within these five parricide cases, two involved daughters killing their mothers, both of whom had a mental illness that was deemed to be legally causative of the killing. The third involved a son–father killing, again involving a mental illness that was deemed to be legally causative of the killing. The fourth involved a son–mother killing, where there was evidence of mental illness, but the perpetrator took his own life and so there was no legal verdict regarding the mental illness being legally causative. And the fifth case involved a transwoman killing her father, who had a mental illness, but this was not deemed to be legally causative. In all of these cases, the parent-victim was reliant upon their son or daughter to provide care, often isolated and otherwise unsupported, and the reviews identify neglect and suspected abuse that was not acted upon. This subtype of parricide resonates with Bojanic et al.’s (2020) category of “middle-aged with affective disorder” perpetrators, who were commonly caring for their aging and ailing parents, and suggests a need for further research into this context of parricide, which also falls within our theme of parental proximity.

## Discussion

### The Role of Mental Illness, Gender, and “Parental Proximity” in Parricide

The centrality of the victims in both the lives and care of the perpetrators was a consistent theme running throughout cases where mental illness, and schizophrenia, in particular, was found to be legally causative in the parricide. Indeed, the victims themselves were often plugging the holes in the provision of clinical care for perpetrators at the time of the killing. Adults with mental health conditions such as schizophrenia often require a high level of support and, in many of these cases, the mental illness was seriously detrimental to the perpetrator’s life and they were unemployed, single, and without children, largely isolated, and dependent on the support of their parent.

The theme of parental proximity reflects a common thread that underscored most of the cases in the dataset: Parent victims were subject to a “double bind” of responsibilization and marginalization in the care of their mentally ill adult child, and this was particularly apparent in the matricide cases. On one hand, mothers were overburdened with caring responsibilities which included providing a home, financial support, overseeing health care, emotional and social support, and advocating with a range of services, sometimes with the support of other family members, but frequently alone and in the absence of their own network of support. There were also assumptions (highlighted within the DHRs) that mothers would fulfill this role, made by mental health and medical services. For example, one DHR noted that it had been wrong to expect a mother who was eventually killed to be responsible for monitoring her son for increased severity of psychotic symptoms and to be responsible for alerting mental health services if this happened. In another DHR it was noted that a mother should not have been given responsibility for ensuring her son adhered to a treatment plan when she herself was frightened of him. On the other hand, they were kept at arm’s length in the treatment of their adult child and their concerns were not taken seriously.

The responsibilization of parents generally, and mothers specifically, for the conduct and care of their children is an enduring social phenomenon (Condry & Miles, 2012), and the burdens placed on parents in this context could be seen as an example of the wider sociopolitical forces of “neo-liberal responsibilization,” which renders individuals and families responsible for tasks which might previously have been the duty of state agencies (Garland, 1996). A retraction of state welfare, support, and services such as mental health, is coupled with a clear agenda of individual and familial responsibility, locating responsibility for care needs within the family, and particularly burdening women who are expected to “take up the slack” of the retraction of state-provided services such as health and social care (Bakker, 2007, p. 546). This is characterized by a “progressive detachment of individuals from social networks and supports, while at the same time, responsibility for systemic problems is being downloaded onto the individual” (Brodie, 2003, p. 67), which can include shifting responsibility for managing risk to individuals (O’Malley, 1996). The impact of a gendered neoliberal political economy on family life is a wider societal phenomenon, but our data illuminate how problematic and potentially dangerous this becomes when intersecting with the increased risk of violence/homicide associated with severe mental illness and processes which responsibilize families in monitoring and managing this risk.

The burden of care in our cases fell particularly to mothers. We refer above to “parental” proximity because there are cases where fathers were killed by their mentally ill son/daughter. However, it was an overwhelming finding in the matricide cases. Of the 20 cases involving mentally ill perpetrators for which we were able to access a full case review, 14 parent-victims were the primary carer for the perpetrator and 10 of these parents were mothers. This occurs within an ideology of “intensive mothering” (Hays, 1996), which increases the expectations and workload of mothers and the pressure to cultivate successful children and blames them if their children do not succeed (O’Reilly, 2014). Themes of mother-blame are strong in the contemporary world across a range of domains (Caplan, 2013) and have a long history in psychiatry with the concept of the “schizophrenogenic mother” popular until the 1970s (Harrington, 2012; Neill, 1990).

As we have noted, responsibilization of parents, and particularly of mothers, occurs simultaneously with their marginalization in the treatment of their adult son/daughter and a lack of recognition of their own support and safeguarding needs. Common themes in the DHRs and other reviews across the cases in our study included:

- Failure of services to take the concerns of the parent-victim and other family members seriously;
- Not understanding the parent-victim as a victim of domestic violence (or potential victim) and the particular dynamics of adult child to parent violence, including not investigating previous injuries;
- Failure to ensure the perpetrator received appropriate treatment; failure to properly assess risk or see the parent-victim as a person in their own right in need of safeguarding;
- Failure to provide proper information to family members about the perpetrator’s illness to help them assess their own risk;
- Failure to ensure family members were supported or to offer them caring assessments; poor record keeping and sharing of information between services; missed opportunities, mistakes, and unsound decisions which proved to be calamitous.

We repeatedly found examples of family members not being consulted in the treatment of the mentally ill perpetrator, not informed about their diagnosis or treatment, and not listened to when they expressed concerns. This marginalization extended to not recognizing the serious risk to the parent-victim or identifying their need for support.

The theme of parental proximity also appeared to extend to circumstances where the mentally ill perpetrator was acting in a carer capacity for the parent-victim; within the subset of 21 cases for which we were able to access full reviews, five cases involved this context. The expectation on sons/daughters to care for elderly and unwell parents could therefore be understood to represent the reverse side of the “normative coin” when considering the intersection between proximity and psychiatric ill-health in parricide.

The role of “caring” has been identified as a risk factor for fatal violence in current/former partner and familial homicides in recent studies of DHRs; Benbow et al. (2019) highlight how “being cared for” can be stressful for both the carer and person receiving the care and also note how parents caring for their mentally ill adult-aged son/daughter can be rendered vulnerable. They similarly found that parent-carers were placed at risk through not being sufficiently involved in the care-making decisions surrounding their children, despite their integral role to their son/daughter’s care. Sharp-Jeffs and Kelly (2016:63) emphasize the vulnerability of parents with “caring responsibilities,” citing two cases in which fathers were killed by their sons, who had “known and significant mental ill health.” Their recommendations include carrying out carer assessments and involving parent-carers in the construction of care plans, as well as enacting safeguards for parents caring for their mentally ill adult-aged sons/daughters.

These findings closely resonate with ours, which highlight both the high levels of mental illness among perpetrators of parricide, and the broader context of “parental proximity.” Severely mentally ill adults (mostly sons) frequently cared for by their parents (primarily mothers), especially in times of austerity, where mental health services are overstretched and underresourced, and in the context of a long-term retraction of mental health services. This leads to a situation in which parents are responsibilized and integral to their care, and relied upon to continue providing this, even during times of crisis and when feeling vulnerable and concerned for their own and/or their son/daughter’s safety. Likewise, the reverse also appears to be true in cases where vulnerable sons and daughters (who may themselves require clinical support) are relied upon to provide care to elderly/unwell parents. This has the inevitable consequence of placing already vulnerable parents within the immediate (and often, due to the cared-for context, inescapable) proximity of harm.

## Conclusion

The killing of parents by children of all ages, known as parricide, is an underresearched form of violence and homicide. From a criminological perspective, the major body of literature derives from the seminal work of Kathleen Heide and colleagues over the past three decades. Heide’s (2013a) typology of parricide, based on parent killings in the United States, has provided invaluable insight into the contexts of parricides and highlighted the connections between parricide and severe mental illness, dangerous antisocial personality disorder, and histories of child abuse. However, the social, cultural, health, and welfare context in the United States is distinctive from the UK, and as we have argued here it is necessary to develop culturally specific analyses of parricide to build an international body of criminological work.

The most striking finding from our analysis of 57 parricide cases related to the high preponderance of mental illness among parricide perpetrators, supporting Bojanic et al.’s (2020) recent findings and broader literature on domestic homicide and femicide. In particular, we found support for the relationship between diagnoses of schizophrenia and other delusional disorders, although other mental illnesses such as depressive disorders and comorbid substance misuse were also identified. In total, there was evidence of mental illness diagnoses for 74% of the 57 parricide offenders and in 79% of these cases, the mental illness was found to be *legally causative*.

Our analysis points to the inadequacies of HI data which is reflective of broader challenges in accessing accurate and detailed data on parricide that enable nuanced understandings and prevention strategies to be developed. Our findings on high rates of mental illness support those of a quantitative study that supplemented HI data with mental health services data, finding that over two-thirds of parricide offenders had mental illness diagnosed (Bojanic et al., 2020). A handful of publications focusing on domestic homicide and femicide in the UK have drawn attention to mental illness as a key contextual factor in the small number of parricides included in their data (e.g., Benbow et al., 2019; Bows, 2019; Brennan, 2016; Chantler et al., 2020; Montique, 2019). Despite the limitations of HI data, it is considered a robust source of data and provides a useful insight into the extent, nature, and prevalence of parricide and has been used here to examine broad patterns and trends in contemporary parricide across England and Wales (2003–2016).

Our exploration of the mechanisms for this correlation revealed a core theme of “parental proximity,” characterized by parent-victims (often mothers) providing full-time care for their mentally ill adult son/daughter. The broader context surrounding this parental proximity stems from a shift from institutional to community care over the past 30 years, alongside increasing cuts to mental health services and a reliance on family members to provide care for their loved ones with sometimes very severe mental illnesses. Middle-aged and elderly parents caring for their adult sons/daughters are often highly marginalized and invisible to services as potential victims, despite the risks of violence they face as a result. This marginalization is rooted in gender and age; further research is needed to explore how it intersects with other structures of inequality such as race, social class, disability, and sexuality. There was evidence in the case study dataset of parents being simultaneously responsibilized for their son/daughter’s care, at the same time as being excluded from care assessment plans. Importantly, we also found a different form of parental proximity in a number of cases in which parent-victims were killed by their mentally ill son or daughter who was caring for them. Overall, the intersection of service withdrawal, parental proximity, and mental illness, appears to contextualize a substantial number of parricides.

Our analysis provides further support to previous research indicating that not only is parricide highly gendered—women are overrepresented as victims and the overwhelming majority of parent-killings are perpetrated by sons—but also, that mother killings (matricides) and father killings (patricides) have distinctive characteristics and circumstances. Our closer analysis of matricide cases found that mothers were frequently the primary carer to their severely mentally ill son, often the “last woman standing” in providing this care, their own needs and vulnerabilities disregarded. Applying a feminist gaze to the deeper contexts of parricide reveals it as primarily male perpetrated violence, experienced differently by men and women, and matricide as constructed around gender norms of caring and responsibilization, coinciding with the marginalization and disempowering of women.

Prevention strategies need to recognize the risk of serious mental illness to parent carers (and in some cases, parents being cared for), their isolation, and their need for support. Services need to work collaboratively and inclusively with parents providing support to mentally ill adult sons/daughters while not overburdening them with responsibility. There is a need to recognize the considerable caring burden placed on parents caring for adult sons/daughters with serious mental illness and to identify parents providing this care as carers in need of support. NICE^10^ guidelines state that carers’ assessments should be offered to those providing care so additional supports can be considered such as a carer’s allowance, psychological and family interventions (NICE 2020), and in most cases in this study, they were not offered to parents. Potential risk to parents must be fully understood by mental health services, with training to identify signs of adult child-to-parent domestic abuse and referrals made to specialist domestic abuse services. It is important that parents are not overburdened or exposed to heightened risk by decisions about treatment and care, such as releasing a patient deemed high risk to live alone with his mother, or depending on a mother to monitor and enforce a treatment program. Similarly, domestic abuse services would benefit from increased knowledge and training to understand how the risk of violence from adult children intersects with mental illness and prevention and support measures might be tailored to the child–parent relationship.

A better evidence base on adult-aged child-to-parent domestic abuse, parricide, and the gendered dynamics of filial violence, will be critical to the prevention of parricide. The study of parricide needs to build upon the insights of decades of work on male violence against women. Feminist sociological analyses will help us to better understand the wider societal and cultural processes that create the circumstances in which parricides occur, to understand the continuities between parricide and other forms of male-perpetrated familial violence, and to fully explore its intersection with ideologies of gender, caring, and motherhood.

## Notes

1. Homicide Act 1957 (as amended by the Coroners and Justice Act 2009), s.2

2. Homicide Act 1957 (as amended by the Coroners and Justice Act 2009), s.2(1A)

3. Homicide Act 1957 (as amended by the Coroners and Justice Act 2009), ss.2(1)(c) and 2(1B)

4. See for example, Sentencing Council, “Sentencing Offenders with Mental Health Conditions or Disorders—for consultation only,” https://www.sentencingcouncil.org.uk/offences/magistrates-court/item/sentencing-offenders-with-mental-health-conditions-or-disorders-for-consultation-only/

5. Mental Health Act 1983, S.45a

6. See, for example, Sentencing Council ,“Sentencing Offenders with Mental Health Conditions or Disorders—for consultation only,” https://www.sentencingcouncil.org.uk/offences/magistrates-court/item/sentencing-offenders-with-mental-health-conditions-or-disorders-for-consultation-only/

7. Domestic Homicide Reviews established on a statutory basis in England and Wales under the Domestic Violence, Crime and Victims Act 2004, but only became a statutory requirement in April 2011.

8. In 2013, NHS England assumed overarching responsibility for the commissioning of independent investigations into mental health homicides and serious incidents and introduced its revised Serious Incident Framework in April 2015. IIMHH reports follow an independent investigation into the care and treatment received by a patient with the objective of learning lessons for future prevention.

9. A multiagency review that is conducted by a Local Safeguarding Adults Board under the 2014 Care Act to determine what agencies or individuals could have done to prevent serious harm or death taking place.

10. The National Institute for Health and Care Excellence, an executive nondepartmental public body of the Department of Health in England, responsible for improving outcomes and upholding standards in the health and social care system.
